# Development of Single-Walled Carbon Nanotube-Based Electrodes with Enhanced Dispersion and Electrochemical Properties for Blood Glucose Monitoring

**DOI:** 10.3390/bios14120630

**Published:** 2024-12-19

**Authors:** Dong-Sup Kim, Abdus Sobhan, Jun-Hyun Oh, Jahyun Lee, Chulhwan Park, Jinyoung Lee

**Affiliations:** 1 Department of Green Chemical Engineering, Sangmyung University, 31 Sangmyungdae-Gil, Dongnam-Gu, Cheonan 31066, Republic of Korea; wavekds@naver.com; 2 Department of Agriculture and Applied Science, Alcorn State University, Lorman, MS 39096, USA; abdus.sobhan@jacks.sdstate.edu; 3 Department of Plant and Food Sciences, Sangmyung University, 31 Sangmyungdae-Gil, Dongnam-Gu, Cheonan 31066, Republic of Korea; junhyunoh@smu.ac.kr; 4 Department of Convergence Bio-Chemical Engineering, Soonchunhyang University, 22, Soonchunhyang-Ro, Sinchang-Myeon, Asan-si 31538, Republic of Korea; jhlee84@sch.ac.kr; 5 Department of Chemical Engineering, Kwangwoon University, 20 Kwangwoon-Ro, Nowon-Gu, Seoul 01897, Republic of Korea; 6 Department of Gyedang College of General Education, Sangmyung University, 31 Sangmyungdae-Gil, Dongnam-Gu, Cheonan 31066, Republic of Korea

**Keywords:** dispersity, biosensor, single-walled carbon nanotube, electrical conductivity, sensitivity

## Abstract

The evolution of high-performance electrode materials has significantly impacted the development of real-time monitoring biosensors, emphasizing the need for compatibility with biomaterials and robust electrochemical properties. This work focuses on creating electrode materials utilizing single-walled carbon nanotubes (SWCNTs) and multi-walled carbon nanotubes (MWCNTs), specifically examining their dispersion behavior and electrochemical characteristics. By using ultrasonic waves, we analyzed the dispersion of CNTs in various solvents, including N, N-dimethylformamide (DMF), deionized water (DW), ethanol, and acetone. The findings revealed that SWCNTs achieved optimal dispersion without precipitation in DMF. Additionally, we observed that the electrical resistance decreased as the concentration of SWCNTs increased from 0.025 to 0.4 g/L, with significant conductivity enhancements noted between 0.2 g/L and 0.4 g/L in DMF. In constructing the biosensor platform, we employed 1-pyrenebutanoic acid succinimidyl ester (PBSE) as a linker molecule, while glucose oxidase (Gox) served as the binding substrate. The interaction between Gox and glucose led to a notable decrease in the biosensor’s resistance values as glucose concentrations ranged from 0.001 to 0.1 M. These results provide foundational insights into the development of SWCNT-based electrode materials and suggest a promising pathway toward the next generation of efficient and reliable biosensors.

## 1. Introduction

The development of high-sensitivity biosensors relies heavily on the optimization of carbon nanotubes (CNTs), specifically focusing on enhancing their dispersity and electrochemical properties while maintaining proper electrical conductivity [1,2]. Achieving well-dispersed CNTs is essential to prevent agglomeration, ensuring uniform coverage and accessibility of the nanotube surface for biomolecule attachment [3]. Biosensors developed with well-dispersed and optimized CNTs have the potential to detect biomolecules with enhanced sensitivity and rapidity. Diabetes mellitus is a chronic metabolic disorder affecting millions of people worldwide [4,5,6]. Non-invasive glucose monitoring has emerged as a highly sought-after alternative to blood-based measurements, offering continuous monitoring without the need for repeated finger pricks [7]. The development of reliable biosensors capable of non-invasively detecting glucose in sweat is of significant interest for diabetes management [8].

CNTs have garnered significant attention in scientific studies because of their unique chemical and physical properties. These attributes have inspired researchers to create advanced analytical tools capable of identifying various biomolecules, including nucleic acids, proteins, glucose, and bacterial cells from both prokaryotic and eukaryotic origins [9]. Structurally, CNTs are formed from graphene layers and can exist as single-walled nanotubes (SWCNTs) or multi-walled nanotubes (MWCNTs), depending on the arrangement of the graphene sheets [10].

SWCNTs have emerged as a versatile nanomaterial for biosensing applications, owing to their exceptional electrical, mechanical, and chemical properties [11]. SWCNTs are highly suitable for glucose sensing applications due to their superior electrical conductivity, extensive surface area, and excellent biocompatibility [11,12]. These nanotubes have a diameter typically in the order of nanometers, with lengths ranging from micrometers to millimeters that lead to a wide range of potential new research applications [13]. The discovery of SWCNTs, along with their multi-walled counterparts (MWCNTs), has opened new avenues for innovation and applications in nanotechnology [12]. Multi-walled carbon nanotubes (MWCNTs) have garnered significant attention in the field of biosensor research due to their unique properties, including electrical conductivity, increased surface area, and biocompatibility [14]. Unlike SWCNTs, MWCNTs consist of several nanotubes nested within one another, with the number of layers typically ranging from two to a few dozen [15]. This unique structure imparts distinctive properties to MWCNTs, making them valuable across a wide range of applications in various scientific and industrial fields.

By leveraging the specific interaction between glucose and enzymes immobilized on SWCNT surfaces, the detection of D-glucose can be achieved with remarkable sensitivity and selectivity [12]. The proposed SWCNT-based biosensor offers several advantages for non-invasive glucose monitoring in sweat [13]. Firstly, the high electrical conductivity of SWCNTs enables efficient charge transfer, facilitating sensitive and rapid glucose detection. Secondly, the large surface area of SWCNTs provides ample sites for enzyme immobilization, enhancing the biosensor’s sensitivity and detection limits [8]. Moreover, the biocompatibility of SWCNTs ensures long-term stability and compatibility with sweat constituents, enabling reliable and accurate glucose measurements [14].

Furthermore, the unique mechanical properties of SWCNTs make them suitable for the development of miniaturized biosensor devices, which are highly desirable for point-of-care diagnostics [12]. In this research paper, we highlight the development and characterization of a SWCNT-based biosensor for D-glucose detection in sweat. The fabrication process involves the functionalization of SWCNTs, followed by the immobilization of glucose oxidase (Gox) as the sensing element [15]. The performance of the biosensor is systematically evaluated using standard glucose solutions, as well as sweat samples collected from healthy individuals. The objectives of this study are to determine the dispersity of CNTs in the solvents for biosensor or sensor development; optimize various parameters, including enzyme loading, pH, and temperature, to enhance the biosensor’s performance; and assess or evaluate the response of the biosensor to potential glucose interferents with rapidity and sensitivity.

In addition to the remarkable properties of carbon nanotubes, the use of chitosan, a natural polymer, has been gaining traction in the field of biosensing due to its excellent film-forming ability and biocompatibility [16]. Chitosan offers a favorable microenvironment for enzyme immobilization, significantly affecting the stability and activity of the immobilized enzymes such as glucose oxidase (Gox) [17]. The integration of chitosan with carbon nanotubes has been shown to further enhance the dispersion of CNTs and prevent their agglomeration, thereby maintaining the high surface-to-volume ratio crucial for enzyme attachment and biosensor sensitivity [18].

To further enhance enzyme immobilization and stability on carbon nanotubes, linker molecules such as 1-pyrenebutanoic acid succinimidyl ester (PBSE) have been employed [19]. PBSE acts as a bifunctional agent where its pyrene moiety adheres strongly to the CNT surfaces through π–π stacking interactions, and the succinimidyl ester group covalently binds to amino groups of enzymes like (Gox) [20]. This technique facilitates a more robust and oriented attachment of enzymes onto the CNTs, promoting efficient electron transfer and preserving enzyme activity [21].

## 2. Materials and Methods

### 2.1. Chemicals

SWCNTs and MWCNTs (called CNTs) were supplied by Sigma Aldrich. St. Louis, MO, USA, with a purity greater than 95%. The solvents, namely N, N-dimethylformamide (DMF), acetone, and ethanol, were purchased from Sigma Aldrich (USA), while deionized water (DW) was prepared and used in the laboratory. The glucose substrate (ng/mL, naturally purified and prepared in) and glucose oxidase enzyme for glucose detection were also procured from Sigma Aldrich (USA).

Phosphate-buffered saline (PBS) solution at 10% (*v*/*v*) concentration (0.1 M, pH 7.4, containing 0.8% NaCl) was obtained from Life Technologies (Seoul, Republic of Korea) and prepared by mixing with purified water. The glucose substrate was diluted in this 10% PBS for experimental use. All other chemicals were of analytical reagent grade and were used without further purification.

### 2.2. Solvent Suspension and Dispersion of CNTs

In this study, the commonly used solvents for preparing spinning solutions included DMF, acetone, ethanol, and DW. The dispersion of the CNTs (SWCNTs or MWCNTs) was performed by mixing CNTs with DMF, acetone, ethanol, and DW, individually, at a concentration of 0.5 g/L and sonicated for 2 h for preparing CNTs’ spinning solutions using ultrasonic waves (write the equipment details). After that, the physical properties of dispersed CNTs’ solution, like concentration measurement using a high UV spectrophotometer, were performed to confirm their effective chemical interaction and high reproducibility with the applied reactant spinning solvents.

The dispersion of CNTs was primarily assessed through a sedimentation process by following the previous procedure with minor modifications [16]. The sediment CNTs in the sonicated solution were captured and monitored with a hand-held smart photometer after one day, one week, and a month to observe the long-term stability of the dispersions. UV spectroscopy analysis was performed using a SHIMADZU instrument at 100.0 kV. Additionally, to further understand the interactions between the nanotubes and solvents, UV measurements were conducted. We measured the absorbance of the CNT dispersions over a wavelength range of 400 to 800 nm using a UV–Vis spectrophotometer. Specifically, we focused on the absorbance at 580 nm, which is a characteristic peak for CNTs due to their electronic transitions. Measuring at this wavelength allowed us to accurately assess the dispersion quality and concentration of CNTs in different solvents. These measurements were used to determine the surface charge of the nanotubes in different solvents, which is a critical factor in understanding their dispersion and stability [16]. The comprehensive analysis offered valuable data optimizing solvent choice and processing conditions for MWCNT applications in various fields, including sensor technology, nanocomposites, and electronic devices.

### 2.3. Biosensor Development with SWCNTs

The biosensor was developed with SWCNTs by following a procedure with slight modifications [17]. Firstly, different concentrations of SWCNTs (0.025, 0.05, 0.1, 0.2, 0.4 g/L) were dispersed in DMF solution and sonicated for at least 2 h. DMF was chosen as the dispersing solvent for CNTs due to its strong solvating ability and high polarity, which effectively interact with the hydrophobic surfaces of CNTs to achieve stable dispersions. This will be explained in more detail in the following section. Following sonication, each sonicated SWCNT with an aliquot of 10 µL was applied to the sensor electrode and annealed for 15 min in the incubator at 80 °C to bind the SWCNTs on the sensor electrode. We fabricated the electrodes using a 6-inch Si wafer with an Au pattern process manufactured by SJ Company. The wafer has a thickness of approximately 675 μm. A thin film was prepared by applying 10 µL of the SWCNT dispersion onto the sensor plate (a glass substrate with pre-patterned electrodes). The dispersion was applied to the plate using the drop-casting method and annealed at 80 °C for 15 min to enhance adhesion. The resulting film covers an area of approximately 1 cm^2^. After annealing, the biosensor plates were washed with DW to eliminate unbound SWCNTs and dried with N_2_ gas. Following this, the electrical resistance of the biosensor with respect to each concentration was measured using a multimeter tester to evaluate the impact of SWCNT concentrations on conductivity.

The 1-pyrenebutanoic acid succinimidyl ester (PBSE) solution acts as a linker for the immobilization process between SWCNT and enzyme, which is a critical step for facilitating electrochemical reactions. This functionalization ensures the effective attachment of enzymes onto the nanotubes, enabling efficient electrochemical performance. To optimize PBSE, the developed SWCNT-based biosensor was functionalized with different concentrations of a PBSE linker (1.0, 2.0, 4.0, 6.0, 8.0 g/L) to investigate how the linker concentration affects the overall conductivity and stability of the dispersion. Each concentration was thoroughly tested to determine the most effective ratio of linkers for enhancing the electrical properties of the biosensor. In addition, to optimize Gox as a binding substrate, the linker-functionalized biosensor was carefully immobilized with Gox at varying concentrations (0.01, 0.05, 0.1, 0.2, 0.5 g/L), and its effect on the electrical properties of the composite material was studied. The immobilization of Gox at different concentrations was crucial for determining the optimal amount required to achieve the best sensor performance. To further enhance enzyme immobilization, a solution mixing method was employed, where chitosan was dissolved with the enzyme solution before applying it to the SWCNT-based biosensor, ensuring a uniform and stable attachment of glucose oxidase onto the biosensor surface [22]. The immobilization of Gox at different concentrations was crucial for determining the optimal amount required to achieve the best sensor performance.

### 2.4. Detection of Glucose by the Fabricated Biosensor

The Gox immobilized biosensor was tested using 80 μL of glucose solutions at various concentrations ranging from 0.001 to 0.1 M, prepared under room temperature conditions. The real-time course of 30 min was performed for glucose detection using the developed biosensor. The biosensor enables continuous glucose detection due to the oxidized enzyme (Gox) as a biosensor receptor, unlike an antibody or DNA fragment. Following the reaction, the biosensor was cleaned with DW to eliminate any residual glucose molecules from the sensor surface. The resistance of the sensor electrode was then measured using a multimeter. The change in resistance confirmed the enzymatic oxidation of glucose catalyzed by Gox, leading to electron transfer changing the electrical conductivity. The change in resistance confirms the Gox reaction catalyzed with glucose, where glucose is oxidized to gluconolactone, which rapidly converts to gluconic acid, and oxygen is reduced to hydrogen peroxide. This reaction induces electron transfer, affecting the electrical properties of the sensor and enabling glucose detection. In this experimental setup, resistance measurements were performed at each step using a multimeter (DT9205A, Digital multimeter, Seoul, Republic of Korea) at room temperature conditions.

## 3. Results

### 3.1. Dispersion Characteristics of CNTs in Various Solvents

**Figure 1:**
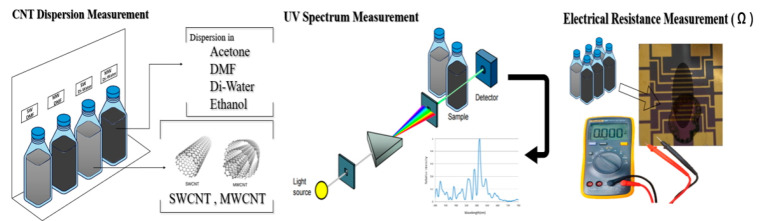
Experimental scheme of the CNT dispersion in various solvents (DMF, acetone, DW, and ethanol) and degree of dispersion measured with UV–Vis and electro resistance.

Figure 1 presents the experimental scheme for the dispersion of CNTs in various solutions, followed by the measurement of dispersion and electrical resistance. The process initiates with the preparation of CNT suspensions in selected solvents, acetone, DMF, DW, and ethanol, under ultrasonication to achieve optimal dispersion. The degree of dispersion is then quantitatively assessed using UV–Vis spectroscopy, where absorbance patterns provide insight into the uniformity and stability of the CNTs within the solvents. Subsequently, the electrical resistance of the dispersed CNTs is measured, offering a functional evaluation that correlates the quality of dispersion with the electrical properties vital for biosensor applications. This schematic illustration encapsulates the critical steps undertaken to ensure the effective integration of CNTs for enhanced performance in biosensor devices. 

### 3.2. Dispersion Analysis of CNTs in Various Solvents

Functionalization of nanomaterials involves altering their chemical and physical properties through chemical reactions [18,23,24]. Optimal interface contact between nanomaterials and reactants is essential for functionalization to ensure effective chemical interaction and high reproducibility of reaction products. Solvents are employed to create a spinning solution that can dissolve the specified polymer materials. However, not all solvents are universally suitable due to their varying abilities to dissolve polymers. Beyond dissolving polymers, solvents play additional roles in the electrospinning process. They aid in maintaining the viscosity of the spinning solution and facilitate the integration of polymer materials with CNTs during electrospinning [22]. Therefore, commonly used solvents for preparing spinning solutions include DMF, acetone, ethanol, and DW.

**Figure 2:**
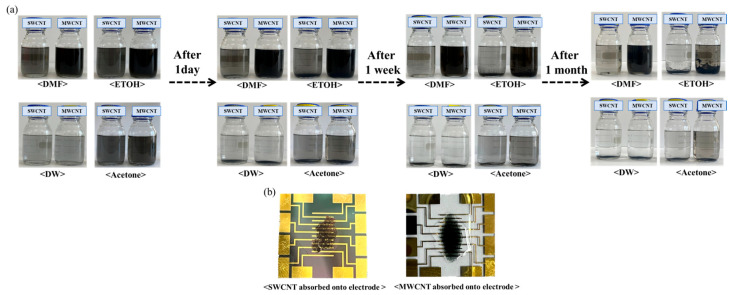
Manufacturing process of biosensor electrode using SWCNTs compared with MWCNTs. (**a**) The visual outcomes of the dispersion process with SWCNTs compared with MWCNTs following a 2 h ultrasonication process in different solvents: DMF, ethanol, DW, and acetone. (**b**) Adsorption process of SWCNTs compared with MWCNTs onto electrodes.

As can be seen in Figure 2, the suspension of SWCNTs shows a uniform and stable dispersion without any visible settling, indicating excellent solubility and stability of SWCNTs in DMF, which can be attributed to the strong interaction between the solvent molecules and the nanotubes [25,26]. Whereas a similarly homogeneous dispersion of MWCNTs was observed in DMF, with the dark coloration suggesting a high concentration of well-dispersed nanotubes [27]. A slight sedimentation at the bottom of the vial indicates a moderate dispersion of SWCNTs in ethanol, reflecting partial compatibility between the solvent and the nanotubes. There is evidence of partial sedimentation; however, the dispersion is notably more homogeneous compared to SWCNTs, suggesting that MWCNTs have a somewhat enhanced interaction with ethanol. Substantial sedimentation into water was observed, indicative of poor dispersion. The lack of stabilization agents in water for SWCNTs leads to significant aggregation. The stark layer separation with sediment at the bottom of the vial confirms the hydrophobic nature of MWCNTs, which do not disperse well in water. The presence of some aggregates amidst a moderately dispersed solution suggests that while acetone can disperse SWCNTs to a certain extent, it is not as effective as DMF. The dispersion is relatively better than that of SWCNTs in the same solvent, which may be due to the larger dimensions and different surface chemistry of MWCNTs, affecting their interaction with acetone. The visual assessment of the suspensions post-ultrasonication provides essential insights into the solubility and stability of carbon nanotubes in various solvents. The observed dispersion levels are crucial for determining the potential application of these nanotube suspensions in the development of biosensors, with DMF being identified as the most suitable solvent for achieving a homogenous dispersion of both types of CNTs.

### 3.3. Morphological Analysis of SWCNTs and MWCNTs via SEM

**Figure 3:**
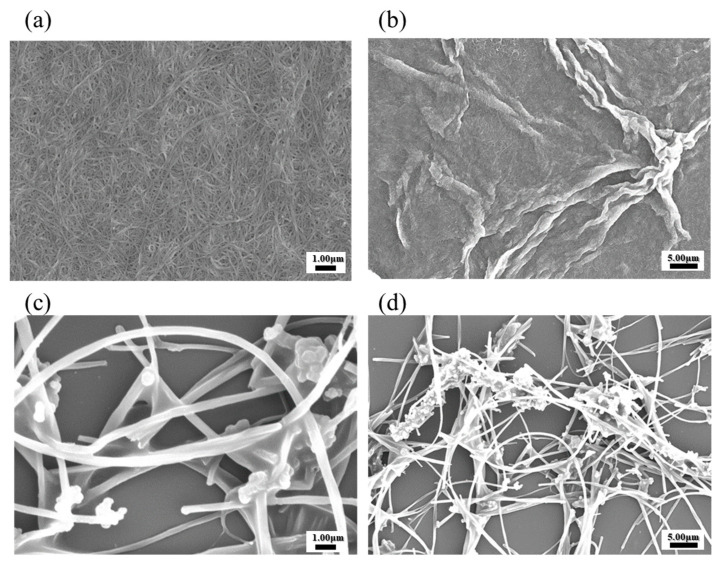
Morphological analysis of SWCNTs and MWCNTs using SEM: (**a**,**b**) SWCNTs; (**c**,**d**) MWCNTs.

The scanning electron microscopy (SEM) images are provided in Figure 3 for the morphological and structural characteristics analysis of both SWCNT and MWCNT [6]. In Figure 3a, SWCNT exhibits a highly uniform and dense network, characterized by an interconnected matrix of nanostructures [18]. This well-organized morphology reflects effective dispersion, particularly in solvents capable of stabilizing the CNTs, such as DMF solvent. The high-resolution image in Figure 3b reveals the fine SWCNTs, with minimal aggregation observed, highlighting the preservation of nanoscale integrity. In contrast, the SEM images of MWCNTs in Figure 3c,d reveal a less cohesive structure with regions of aggregation distributed throughout the network. The larger dimensions and multi-walled configuration of MWCNTs are highlighted by the entangled and densely packed arrangements observed in the magnified image of Figure 3d. These compacted formations are due to weaker solvent interactions, contributing to partial aggregation under the SEM conditions. The structural differences between SWCNTs and MWCNTs in the SEM image were observed to be intrinsically connected to their dispersion behavior [24]. SWCNTs exhibit enhanced uniformity and stability, making them advantageous for applications requiring consistent nanoscale dispersions. MWCNTs, on the other hand, need the additional processing steps and stabilizing agents to achieve similar morphological uniformity, potentially limiting their electrochemical efficiency in certain applications [18].

### 3.4. UV–Visible Spectral Analysis of CNT Dispersions

**Figure 4:**
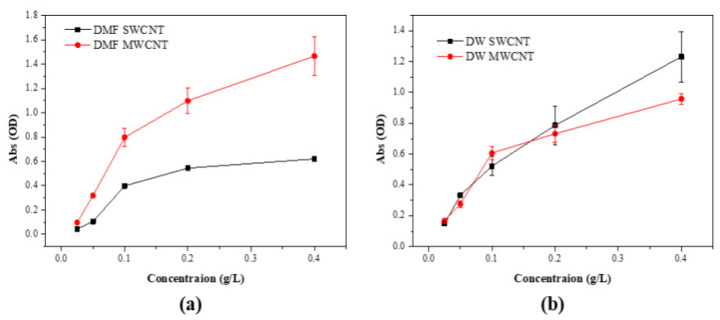
UV–Vis spectrum of DMF and DW aqueous dispersions of the (**a**) SWCNT and (**b**) MWCNT.

The UV–Visible spectral data for dispersions of SWCNTs and MWCNTs in DMF and DW are presented in Figure 4. For SWCNTs in DMF, the spectrum exhibited a progressive increase in absorbance with a rising concentration, indicative of a stable and uniform dispersion. The consistency of the data points suggests effective interaction between the DMF molecules and SWCNTs, facilitating a reliable dispersion suitable for subsequent applications [28]. In contrast, the MWCNTs in DMF showed a similar trend with a slightly increased variability in absorbance, potentially due to the more complex morphology of MWCNTs affecting the uniformity of the dispersion. The SWCNT dispersions in DW demonstrated less steep stability compared to MWCNT in DW. This outcome suggests that without the aid of surfactants or stabilizing agents, DW alone does not provide sufficient dispersal capacity for SWCNTs. MWCNT dispersions in DW also exhibited an upward trend in absorbance, but the slope was less marked compared to SWCNTs, reflecting the inherent difficulty in achieving homogeneous dispersion in a polar solvent like water for these hydrophobic nanomaterials. The spectral analysis underscores the significant influence of solvent choice on the dispersion quality of CNTs. DMF proved to be a superior dispersant for both SWCNTs and MWCNTs, as evidenced by the higher and more consistent absorbance values. This finding is critical for the preparation of CNT-based materials where dispersion quality directly impacts the performance, particularly in sensor technology and nanocomposite fabrication [28,29].

### 3.5. Quantitative Analysis of Electrical Resistance in CNT Dispersions

**Figure 5:**
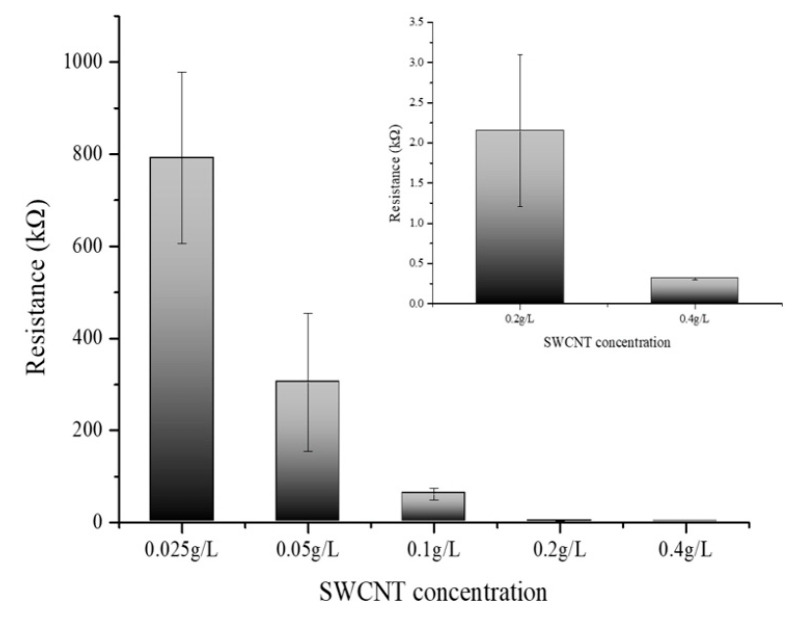
Electrical resistance measurement of different SWCNT concentrations (0.025, 0.05, 0.1, 0.2, and 0.4 g/L).

Figure 5 delineates the variation in electrical resistance as a function of SWCNT concentration. The SWCNT solutions were prepared at concentrations of 0.025 g/L, 0.05 g/L, 0.1 g/L, 0.2 g/L, and 0.4 g/L, and their corresponding electrical resistances were meticulously measured. The data manifest a clear inverse relationship between the concentration of SWCNTs and electrical resistance; as the concentration of the SWCNT solution was augmented, a concomitant decrease in resistance was observed [30,31]. This trend reinforces the assertion that SWCNTs exhibit high electrical conductivity and that an increase in the concentration of these nanotubes facilitates a more efficient electron flow. Notably, the graph illustrates a tapering in the rate of decrease in electrical resistance at concentrations exceeding 0.4 g/L. This concentration suggests an onset of particle-particle interactions where the proximity of SWCNT particles at higher concentrations leads to a saturation point beyond which the resistance reduction is no longer substantial. The phenomena observed postulate the presence of an optimal concentration range wherein the conductive pathways are maximized before the effects of aggregation or inter-tube resistance become dominant. These findings are instrumental in delineating the concentration-resistance profile of SWCNT solutions and have significant implications for their application in nanoelectronics. The results elucidate the potential for tailoring the electrical properties of SWCNT-based materials by controlling their concentration, which is crucial for the development of nanoelectronics devices and circuits. The optimal concentration of SWCNTs for electrical conductivity appears to be at or slightly below 0.4 g/L. At this concentration, the SWCNT dispersion achieves a balance between maximizing conductivity and preventing the onset of counterproductive particle aggregation. Beyond this concentration, the incremental benefits in conductivity diminish, indicating a threshold where further increases in SWCNT concentration do not significantly enhance the electron flow. Thus, for applications requiring high conductivity without the drawbacks of excessive nanotube aggregation, maintaining the SWCNT concentration around 0.1 to 0.4 g/L is advisable. All experiments were performed at least three times to ensure reproducibility and reliability of the results.

### 3.6. Electrical Resistance Variation with Developed Biosensors with PBSE Concentrations

**Figure 6:**
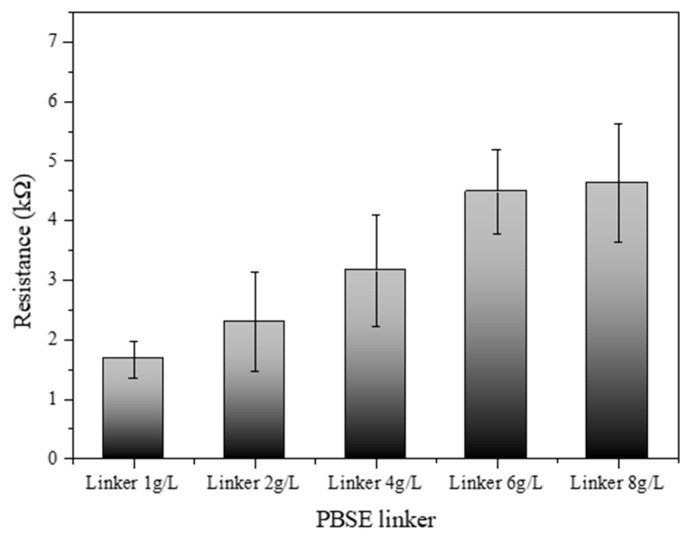
Resistance of SWCNT-based biosensor reacted with different PBSE linker concentrations (1.0, 2.0, 4.0, 6.0, and 8.0 g/L).

Figure 6 illustrates the electrical resistance profile of an SWCNT-based biosensor as a function of varying concentrations of a PBSE linker. The biosensor’s resistance was systematically measured across a series of PBSE linker concentrations: 1.0, 2.0, 4.0, 6.0, and 8.0 g/L. The results demonstrate a discernible increase in resistance with escalating concentrations of the PBSE linker. At the lowest concentration (1.0 g/L), the biosensor exhibited the lowest resistance, suggesting a minimal barrier to electron flow within the SWCNT network. As the concentration of the PBSE linker was increased to 2.0 g/L and 4.0 g/L, a gradual increase in resistance was observed, which may be attributed to the increased density of the linker molecules within the biosensor matrix, possibly leading to more hindered electron mobility. Interestingly, at higher concentrations of 6.0 g/L and 8.0 g/L, the resistance values show a more pronounced escalation, indicating that the PBSE linker concentration has surpassed an optimal threshold for electron transport. This could be due to the formation of a more congested network, where the PBSE linkers introduce additional scattering points for electrons, thus raising the biosensor’s overall resistance [32,33]. These findings elucidate the impact of PBSE linker concentration on the electrical properties of SWCNT-based biosensors. The observed trend highlights the importance of optimizing the concentration of PBSE linkers to achieve desired electrical performance, which is crucial for the sensitivity and specificity of the biosensor applications. The optimal concentration of the PBSE linker appears to lie below 4.0 g/L, where the resistance values begin to rise sharply, suggesting that concentrations above this level might compromise the electrical conductivity of the biosensor.

### 3.7. Optimization of Gox Concentration with Developed Biosensors

**Figure 7:**
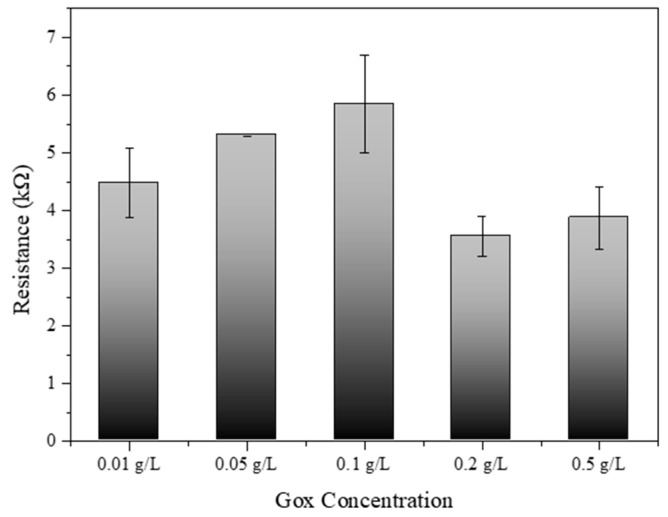
Resistance of SWCNT–PBSE biosensor reacted with different concentrations of Gox (0.01, 0.05, 0.1, 0.2, and 0.5 g/L).

Figure 7 provides significant insights into the optimization of glucose oxidase (Gox) concentration for SWCNT-PBSE biosensors. The experimental data depicted a clear trend in electrical resistance associated with varying levels of Gox concentration. Initially, an increase in Gox concentration from 0.01 g/L to 0.1 g/L corresponded to a rise in electrical resistance. This increment may be due to the insulating properties of Gox, which, when in higher quantity, could hinder the electron transfer across the SWCNT network. Interestingly, a peak resistance was observed at 0.1 g/L, after which the resistance began to decrease at concentrations of 0.2 g/L and further reduced at 0.5 g/L. This phenomenon suggests that there is a critical concentration threshold, where the catalytic activity of Gox begins to outweigh its insulating effects, thus enhancing the overall conductivity of the biosensor. The decrease in resistance at higher Gox concentrations could be indicative of more efficient enzymatic turnover leading to improved charge transfer within the biosensor matrix [33,34]. Therefore, the results from this study suggest that the concentration of Gox plays a pivotal role in the electrical behavior of SWCNT-PBSE biosensors. For applications requiring precise electrical performance, careful calibration of Gox concentration is essential. The optimal concentration for Gox appears to be beyond 0.1 g/L, where the biosensor system benefits from the enzymatic activity without significant compromise to conductivity. Notably, the biosensor exhibited the most efficient performance at a Gox concentration of 0.2 g/L, where the balance between enzymatic activity and conductivity was most favorable. These findings have substantial implications for the design and development of biosensors, particularly in the field of bioelectronics where sensor sensitivity and specificity are paramount.

### 3.8. Glucose Concentration Impact on Electrical Resistance in the Developed Biosensors

**Figure 8:**
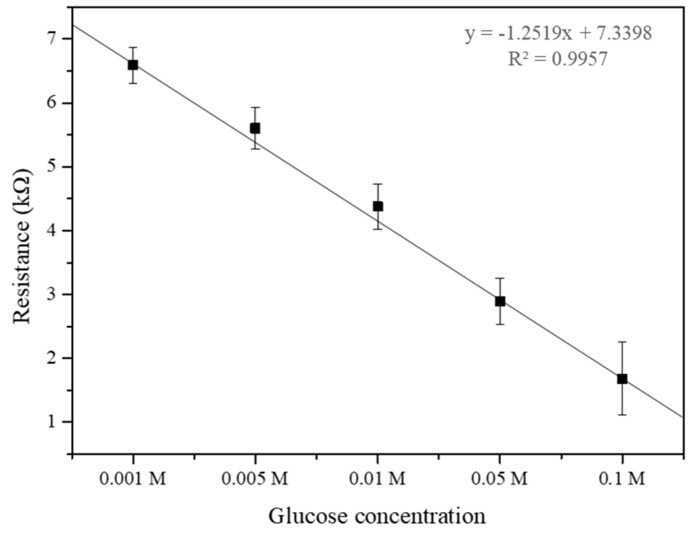
Measurement of electrical resistance sensing different glucose concentrations (0.001, 0.005, 0.01, 0.05, and 0.1 M).

Figure 8 demonstrates the relationship between glucose concentration and the electrical resistance of the SWCNT-based biosensor. The biosensor’s sensitivity to varying glucose concentrations (0.001, 0.005, 0.01, 0.05, and 0.1 M) was quantitatively analyzed through resistance measurements. The experimental data reveal a distinct correlation where the electrical resistance decreases as glucose concentration increases from 0.001 M to 0.1 M. This decrease in resistance likely results from the enzymatic activity of glucose oxidase, which facilitates electron transfer processes in the presence of glucose, thereby enhancing the conductivity of the SWCNT. At higher glucose concentrations of 0.05 M and 0.1 M, the resistance continues to decrease, indicating the biosensor maintains effective enzymatic activity and electron transfer within this range. This saturation could be attributed to the enzymatic reaction approaching its maximum rate, beyond which additional glucose does not correspond to an increase in electron transfer rate. This trend indicates that the SWCNT-based biosensor possesses an optimal detection range for glucose, which is crucial for its application in medical diagnostics, particularly for blood glucose monitoring in diabetic patients [34]. The data from this study suggest that the biosensor maintains high sensitivity and specificity within the lower concentration range (0.001 to 0.1 M), which is within the physiologically relevant range for glucose in human blood. The understanding of this concentration-dependent response is vital for the design of SWCNT-based biosensors, ensuring that they operate within their most responsive range for accurate and reliable glucose detection [34,35]. These results underscore the potential of SWCNT-based biosensors to serve as effective tools for glucose monitoring, with significant implications for the management of diabetes and other conditions characterized by alterations in blood glucose levels.

**Figure 9:**
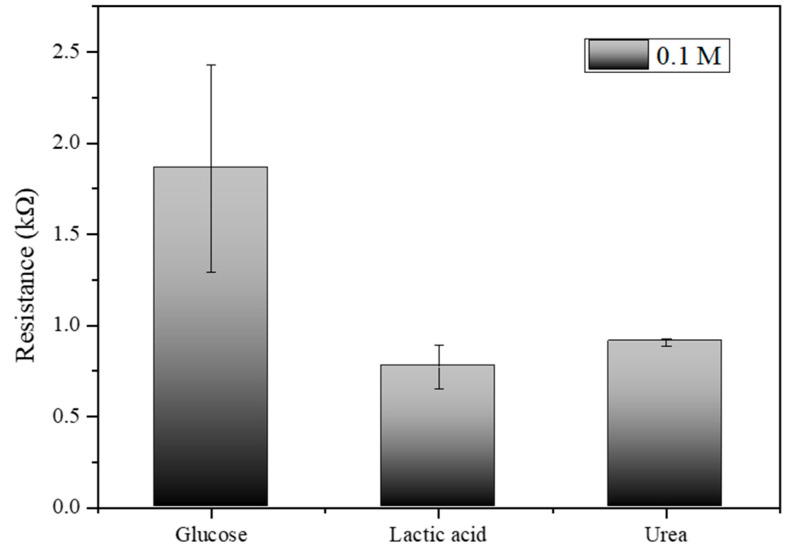
Electrical resistance measurements for glucose, lactic acid, and urea at 0.1 M concentration.

Figure 9, which illustrates the electrical resistance measurements for glucose, lactic acid, and urea at a concentration of 0.1 M, the results indicate that the biosensor exhibits distinct and measurable responses to each analyte. Glucose shows the highest resistance at approximately 1.5 kΩ, lactic acid exhibits a moderate resistance around 0.7 kΩ, and urea displays the resistance close to 0.9 kΩ. The error bars indicate the variability and standard deviation across multiple trials, highlighting the precision of the measurements. These distinct resistance values confirm the biosensor’s capability to differentiate between glucose, lactic acid, and urea, underscoring its specificity and potential effectiveness in glucose detection applications.

In Table 1, various electrodes such as SWCNT-Gox, Au–Nicoaxialnanorad array/GOx, PANI-SDS-F127 (1:1)/GOx, GOx/PVA-Fe_3_O_4_/Sn, and GOx/PtNP/PANI/PtE are presented along with their respective substrates, linear ranges, detection limits, and references. Each entry illustrates the effective use of different nanostructures and polymers in glucose biosensors, showcasing the range of detectable glucose concentrations and the sensitivity limits achieved. The studies highlight the dependence of sensor performance on specific conditions like pH, temperature, and humidity, which are critical for the optimal functioning of these biosensors.

## 4. Discussion

The dispersion quality of carbon nanotubes (CNTs) in different solvents is crucial for their effective use in biosensor devices. Our study found that dimethylformamide (DMF) is the most efficient solvent for dispersing both single-walled carbon nanotubes (SWCNTs) and multi-walled carbon nanotubes (MWCNTs). The strong interactions between DMF molecules and CNTs lead to uniform and stable dispersions, as confirmed by visual assessments and UV–Vis spectral analyses. This high-quality dispersion is essential for applications that require a homogeneous distribution of nanotubes, such as the fabrication of sensitive and reliable biosensors.

In contrast, solvents like ethanol, acetone, and DW were less effective in dispersing CNTs. While ethanol and acetone provided moderate dispersion for MWCNTs, they were less successful with SWCNTs, likely due to differences in surface chemistry and dimensions that affect how the solvents interact with the nanotubes. The poor dispersion observed in DW highlights the hydrophobic nature of CNTs and emphasizes the need for surfactants or functionalization methods to improve their solubility in aqueous environments.

This work performed dispersion tests only on the two types of nanotubes and conducted the remaining experiments using SWCNTs, which have better electrochemical efficiency. Figure 2b shows the deposition of SWCNT onto the electrode films, compared with MWCNT. These SWCNT and MWCNT images absorbed onto the electrode illustrated the dispersion quality and uniformity of the nanotube films on the electrodes. Additionally, we have performed SEM analysis to confirm the structure of SWCNTs and MWCNTs after the absorption process (Figure 3). The SEM images provided the detailed visualization of the morphology and confirmed the dispersion and thickness uniformity of the CNTs mediator. The SEM analysis shows that SWCNT films are uniformly dispersed and form a continuous link across the electrodes, whereas MWCNT films exhibit aggregation. Regarding the MWCNT devices, although MWCNTs disperse well in DMF similarly to SWCNTs, their aggregation with larger diameters and multi-layered structures results in low efficiency of the CNT absorption process. In addition, their aggregated structure produces the gap space between the electrodes and biomaterial setup, which hinders the electrical performance of the devices. Consequently, we focused our electrochemical analysis experiments on SWCNT, instead of MWCNT. Electrical resistance measurements demonstrated a clear inverse relationship between SWCNT concentration and electrical resistance.

As the concentration of SWCNTs increased, the electrical resistance decreased, confirming the conductive properties of CNTs. However, this decrease in resistance began to level off beyond a concentration of 0.4 g/L, suggesting that excessively high concentrations may lead to aggregation, which can impede electron flow. Therefore, maintaining SWCNT concentrations between 0.1 g/L and 0.4 g/L is recommended to optimize conductivity while minimizing aggregation. This study also examined the impact of PBSE linker concentration on the electrical properties of SWCNT-based biosensors. Higher concentrations of the PBSE linker led to increased electrical resistance, likely because the insulating nature of the linker molecules hinders electron flow within the CNT network. To preserve the biosensor’s electrical conductivity while ensuring effective functionalization for biomolecule attachment, it is important to optimize the PBSE linker concentration to below 4.0 g/L.

Gox concentration was another critical factor influencing the biosensor’s electrical resistance. We identified an optimal Gox concentration of approximately 0.2 g/L, where the biosensor benefits from enhanced enzymatic activity without significantly compromising conductivity. At this concentration, the catalytic activity of Gox improves electron transfer, enhancing the overall performance of the biosensor. When testing the biosensor’s response to varying glucose concentrations, we observed high sensitivity within the physiologically relevant range for human blood glucose levels (0.01 M to 0.1 M). It will be necessary to present the cyclic behavior of the sensor response to further validate its robustness and ensure practical applicability.

## 5. Conclusions

This study has investigated the dispersion characteristics of CNTs in various solvents, their interaction with PBSE linkers, the optimization of glucose oxidase (Gox) concentration, and the consequent impact on electrical resistance in biosensor applications. The optimal dispersion of CNTs was achieved in DMF, which exhibited the most stable and uniform nanotube suspension, as visually confirmed and quantitatively supported by UV–Vis spectral analysis. The electrical resistance measurements further validated that DMF is the solvent of choice for SWCNT-based biosensors due to its favorable interaction with the carbon nanotubes. The interaction with PBSE linkers and Gox concentration offered a concentration-dependent electrical resistance, which peaked and then plateaued, indicating optimal ranges for biosensor performance. For glucose detection, the SWCNT-based biosensor exhibited enhanced sensitivity at lower concentrations, suggesting its efficacy for blood glucose monitoring within the physiological range. This research underscores the importance of fine-tuning SWCNT, PBSE, and Gox concentrations to achieve biosensors with optimal electrical properties for medical diagnostics. This study features the SWCNT-based biosensor’s potential for accurate and reliable blood glucose monitoring, which is essential for diabetes management. Controlling the concentration of SWCNTs, PBSE linkers, and Gox is critical for optimizing the electrical properties of SWCNT-based biosensors. These insights pave the way for the design of highly sensitive and specific biosensors, which are of paramount importance in the fields of medical diagnostics and bioelectronics.

## Figures and Tables

**Table 1:** Comparison of enzymatic electrochemical biosensors for glucose detection.

Electrode	Substrate	Linear Range (mM)	Detection Limit (uM)	Ref
SWCNT-Gox	Glucose	1.0–100	10.0	This study
Au–Nicoaxialnanorad array/GOx	Glucose	0.0275–27.75	5.5	[36]
PANI-SDS-F127 (1:1)/GOx	Glucose	5.0–50	3.2	[37]
GOx/PVA-Fe_3_O_4_/Sn	Glucose	0.005–30	8.0	[38]
GOx/PtNP/PANI/PtE	Glucose	0.01–8.0	0.7	[39]

## Data Availability

The data presented in this study are available on request from the corresponding author.
